# Downregulation of Integrin β4 Decreases the Ability of Airway Epithelial Cells to Present Antigens

**DOI:** 10.1371/journal.pone.0032060

**Published:** 2012-04-24

**Authors:** Chi Liu, Xiaoqun Qin, Huijun Liu, Yang Xiang

**Affiliations:** Physiology Department, Xiangya Medical School, Central South University, Changsha, Hunan, People's Republic of China; French National Centre for Scientific Research, France

## Abstract

Airway epithelial cells have been demonstrated to be accessory antigen presentation cells (APC) capable of activating T cells and may play an important role in the development of allergic airway inflammation of asthma. In asthmatic airways, loss of expression of the adhesion molecule integrin β4 (ITGB4) and an increase in Th2 inflammation bias has been observed in our previous study. Given that ITGB4 is engaged in multiple signaling pathways, we studied whether disruption of ITGB4-mediated cell adhesion may contribute to the adaptive immune response of epithelial cells, including their ability to present antigens, induce the activate and differentiate of T cells. We silenced ITGB4 expression in bronchial epithelial cells with an effective siRNA vector and studied the effects of ITGB4 silencing on the antigen presentation ability of airway epithelial cells. T cell proliferation and cytokine production was investigated after co-culturing with ITGB4-silenced epithelial cells. Surface expression of B7 homologs and the major histocompatibility complex (MHC) class II was also detected after ITGB4 was silenced. Our results demonstrated that silencing of ITGB4 resulted in impaired antigen presentation processes and suppressed T cell proliferation. Meanwhile, decrease in Th1 cytokine production and increase in Th17 cytokine production was induced after co-culturing with ITGB4-silenced epithelial cells. Moreover, HLA-DR was decreased and the B7 homologs expression was different after ITGB4 silencing. Overall, this study suggested that downregulation of ITGB4 expression in airway epithelial cells could impair the antigen presentation ability of these cells, which further regulate airway inflammation reaction in allergic asthma.

## Introduction

Atopic asthma is characterized by Th2-mediated inflammation and typical impaired airway epithelial cells. In the airways of asthma patients, exposure to allergens induces an increase in Th2 cell infiltration and Th2 cytokine expression [1]. Activated T cells have been implicated in asthma and physically interact with epithelial cells in the airway [2], [3]. As the first barrier to environmental pollutants and allergic stimuli, airway epithelial cells play an important role in driving the activation and differentiation of T cells in respiratory allergies [4], [5]. Several studies have verified that airway epithelial cells can act as effective immune regulatory cells the production of a series of cytokines and chemokines [6], [7]. Recent studies further found that airway epithelial cells can affect the outcome of T cells immune responses by acting as antigen presentation cells (APCs). Effective antigen presentation by APCs is an important way to activate and induce the activation and proliferation of T cells [8], [9]. Airway epithelial cells compose the first cell layer of the respiratory airway and thus are best positioned to identify antigens. Consequently, antigen presentation by airway epithelial cells may be an effective and important way to guard the airway against harmful antigens and to regulate the local T cell immune inflammation in the airway [10].

Inflammation cells infiltration and Th2 biased inflammation are the major phenotypes on the airway of asthma patients [1]. However, the specific mechanism underlying these features is still unclear. Airway epithelial cells from asthma patients often show significant structural damage and loss of functional homeostasis [11], [12], [13]. Loss of structural adhesion could enhance the access of inhaled allergens to underlying APCs. Our previous work demonstrated that structural adhesion molecule integrin β4 (ITGB4) is downregulated in asthma airway epithelial cells. As the first cell barrier to outer allergens, the airway epithelial cells showed a decreased wound repair and anti-oxidation ability after ITGB4 was downregulated [14], [15]. ITGB4 is an important adhesion molecule that mediates the anchoring of airway epithelial cells to the basal membrane. Given that ITGB4 is known to engage in multiple signaling pathways that may influence many physiological functions of airway epithelial cells [16], [17], we questioned whether downregulation of ITGB4 enhanced the invasion of inhaled allergens and regulated the local T cell immune inflammation through antigen presentation process. In this study, ITGB4 was silenced by a specific siRNA virus vector. We observed that downregulation of ITGB4 is associated with blocked antigen presentation ability, decreased MHC class II (HLA-DR) expression and altered B7 homologs expression on airway epithelial cells. Furthermore, decreased T cell proliferation and differential cytokine production was induced after co-culturing with ITGB4-silenced epithelial cells.

## Materials and Methods

### Cell culture

The immortalized human bronchial epithelial cell line 16HBE14o- (kind gift of Prof. Gruenert of the University of California, San Francisco) was maintained at 37°C in 5% CO_2_ in a mixture of DMEM:F12 (1∶1) media supplemented with 100 U/ml penicillin, 100 U/ml streptomycin and 10% heat-inactivated fetal bovine serum (FBS).

### ITGB4-specific siRNA vector construction and transfection

The effective ITGB4 siRNA and control siRNA were annealed and ligated into the new pGCSIL-PUR vector to create an ITGB4 siRNA expression vector and a control expression vector (Genechem, China, Shanghai) [14]. All plasmids were sequenced to confirm that they were correct. Then, the ITGB4 silencing vector (ITGB4 siRNA vector) and nonsense siRNA vector (control vector) were packaged into recombinant lentivirus vectors using Lentivector Expression Systems (GeneChem, Shanghai, China) and titered to 10^9^ TU/mL.

Before transfection, 16HBE14o- cells were plated in 24-well plates (2×10^4^ cells/well) overnight. The lentiviruses were diluted in 0.2 mL (10^8^ TU/mL) of complete medium containing polybrene (8 mg/mL) and added to the cells for 1 h at 37°C, followed by incubation in 0.3 mL of freshly prepared polybrene-DMEM/F12 for another 24 h. Then this media was replaced with fresh DMEM/F12 medium, and the cells were cultured for 48 h. Under our experimental condition, to silence ITGB4, 16HBE14o- was infected with ITGB4-specific siRNA Lentivector vector by addition of lentivirus into the cell culture at a MOI of 5. In this study, the transfection was repeated for each experiment.

### Real-time PCR detection after vector transfection

Real time PCR was used to detect ITGB4 mRNA expression after transfection. The sequences of the primers and taq man probes used are as follows: ITGB4 forward: 5′-CAC CGC GTG CTA AGC ACAT-3′, ITGB4 reverse: 3′ -TGT GGT CGA GTG TGA GTG TTC TG-5′, taqman probe: fam+ACC CTC ACA CGG GAC TAC AAC TCA CTG+tamra, GAPDH forward: 5′ -CCA CTC CTC CAC CTT TGA C-3′, GAPDH reverse: 3′-ACC CTG TTG CTG TAG CCA-5′ and taqman probe: fam+TTG CCC TCA ACG ACC ACT TTGT+tamra. Seventy two hours after transfection, total RNA (1 µg) purified from different vector transfected groups was reverse transcribed into cDNA using AMV reverse transcriptase (Qiagen, Vento, Netherlands) with an RNase inhibitor and oligo d(T) primer at 40°C for 50 minutes followed by heating at 90°C for 5 minutes. Then, 1 µl of the reverse-transcript was added to a 30 µl PCR mixture for 40 cycles. Each cycle included 93°C for 30 s and 54°C for 60 s and used Taq polymerase. Negative controls consisted of an equal volume of water substituted for the volume of RNA in the RT reaction. Normalization of mRNA expression data for sample-to-sample variability in RNA input, RNA quality, and reverse transcription efficiency was achieved by comparing the copy numbers of target mRNAs with that of human GAPDH.

### Western blot analysis after vector transfection

Seventy two hours after transfection, proteins from parental 16HBE14o- cells, control vector transfected cells, and ITGB4 siRNA viral vector transfected cells were separated by SDS-PAGE and then transferred to polyvinylidene difluoride membranes (Millipore, Bedford, MA). Blots were blocked and then probed with antibodies against ITGB4 (1∶1,000 dilution; Santa Cruz, CA, USA) and GAPDH (1∶5,000 dilution; Sigma, St. Louis, MO, USA). After washing, the blots were incubated with horseradish peroxidase–conjugated secondary antibodies and visualized by super ECL detection reagent (Applygen, China, Beijing).

### Antigen presentation detection

To acquire vivid and precise results, the antigen presentation process in 16HBE14o- cells was analyzed by laser confocal microscopy and flow cytometry separately [8].

For laser confocal microscopy detection, 16HBE14o- cells from control group and ITGB4 silence group that adhere to glass coverslips were incubated with the FITC-labeled OVA for various periods; then they were rinsed in PBS and fixed with 3∶1 methanolacetic acid for 5 min at 4°C. Then, fixed cells were blocked with PBS containing 5% goat serum and 0.2% Triton X-100 (Sigma) for 15 min before incubation with the anti-HLA-DR antibody for 1 h. The cells were rinsed three times in PBS before incubation with Dylight 549 (red) labeled conjugated Ig (Thermo Scientific, Waltham, MA). The coverslips containing 16HBE14o- cells were then rinsed in PBS three times and mounted with Immun-mount (Shandon, Pittsburgh, PA) before they were viewed with a laser confocal microscope (Fluorvert, Leica, Deerfield, IL) at a step position of 1 µm on the x-y or x-z axis. Three observers routinely examined 10 separate fields. Mean fluorescence intensities of the internalized FITC-labeled OVA in the cells of the entire field examined were determined with Leica Fluorvert Laser confocal microscope using the image software package.

For flow cytometry detection, cells from different groups were incubated with the FITC-labeled OVA for various periods; then they were rinsed in PBS and collected with PBS containing 1% FBS and 0.01% NaN_3._ The FITC fluorescence intensity of 16HBE14o- cells was analyzed by FACS Calibur flow cytometer (Becton Dickinson, Mountainview, CA).

### Lymphocyte proliferation assay

Peripheral blood was obtained from healthy volunteers following the approval for the use of human tissues was granted by the Ethical Committee of Central South University after obtaining written informed consent from individual donors. Heparinized venous blood was collected and diluted 1∶3 with PBS, layered on a Ficoll-Hypaque density gradient, and centrifuged for 30 min at 1,400 rpm. Peripheral blood T lymphocytes were collected from the interface and washed three times with PBS. Cells were resuspended in RPMI 1640, and cell density was adjusted to 5x10^6^ cells/ml. CD4^+^ T cells were purified from Peripheral blood T lymphocytes by negative selection (Stem-Cell, Vancouver, Canada). Then, 10^5^ CD4^+^ T cells were cocultured with 10^5^ freshly isolated 16HBE14o- cells in triplicate round-bottom plates in 0.2 ml serum-free culture medium at 37°C in a 5% CO_2_ incubator for 3 days with OVA (0.1 μg/ml). To avoid HLA-class II allele unmatching during the co-culturing, all the CD4^+^ T cells from different donors was selected to be matched under our experimental condition. In some experiments ITGB4-silenced 16HBE14o- cells were used to coculture with CD4^+^ T cells. T cells proliferation was assessed by 5-bromo-2-deoxyuridine (BrdU) incorporation assay and carboxyfluorescein diacetate- succinimidyl ester (CFDA-SE) labeling separately.

For CFDA-SE labeling (CellTraceTM CFSE Cell Proliferation Kit, Invitrogen, Eugene, OR, USA), the T cell suspension was washed three times with PBS and finally resuspended in culture medium. T cells were resuspended in PBS at 5×10^6^/ml for staining. CFSE in the form of a 5 mM stock solution in DMSO was added at a final concentration of 1 µM for 10 min at 37°C. The CSFE-labeled cells were cocultured with control transfected cells or ITGB4-silenced cells. After 5 days of coculture, the proliferation of CD4^+^ T cells was detected by assessing CFSE fluorescence intensity.

**Figure 1:**
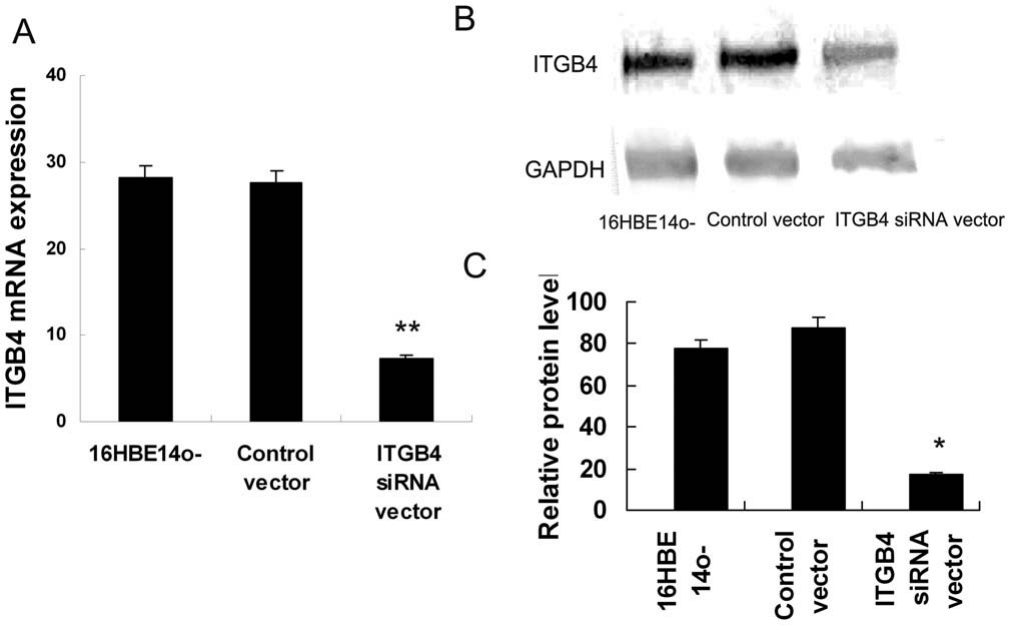
Silencing efficiency was detected after ITGB4 siRNA-containing lentivirus was transfected. The effective ITGB4 siRNA lentivirus vector and control nonsense siRNA lentivirus vector were prepared and transfected into 16HBE14o- cells. (A) 72 h after lentivirus vector transfection, ITGB4 mRNA expression was detected by real time PCR. (B) Western blot analysis was used to detect ITGB4 protein expression 72 h after tansfection. (C) Quantification of western blot normalized to the level of GAPDH in Figure 1A.

For BrdU incorporation (Roche Applied Science, Mannheim, Germany), T cells proliferation was evaluated by measuring BrdU incorporation in a colorimetric assay according to the manufacturer's instructions. The assay was performed according to the instructions of the manufacturer (Roche Applied Science, Mannheim, Germany). Absorbance values were measured at 450 nm using an ELISA reader (Tecan, Crailsheim, Germany). The blank corresponded to 100 ml of culture medium without BrdU. T-cell proliferation was evaluated by measuring BrdU incorporation from the first day to the fifth day after coculture.

**Figure 2:**
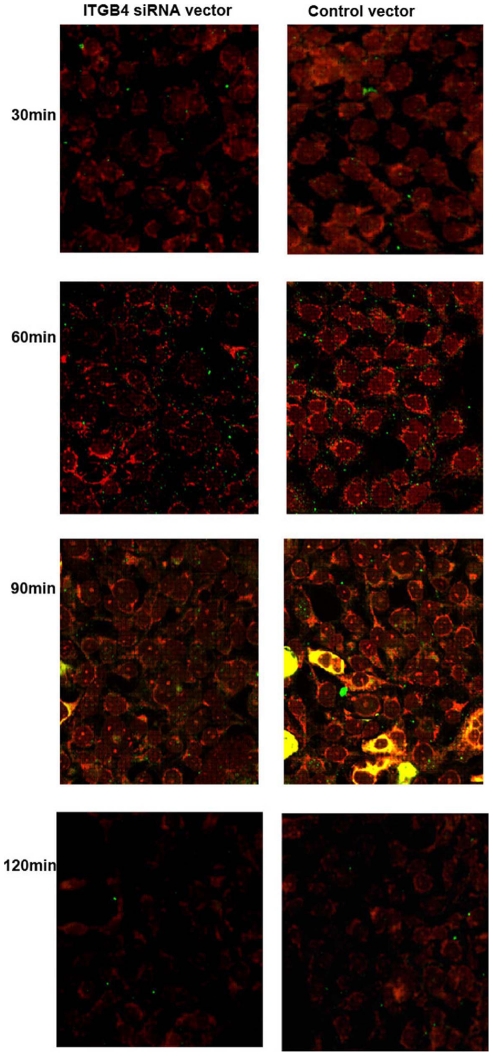
Colocalization of HLA-DR with endocyosed OVA in respiratory epithelial cells. 16HBE14o- cells from different groups were pulsed with FITC-labeled OVA for 0–120 min, fixed, permeabilized, and stained with Dylight 549-labeled anti-HLA-DR antibodies. Cells were analyzed by confocal microscopy. Yellow staining (red+green) indicates colocalization. Results are representative of 1 experiment repeated 3 times.

### ELISA

The levels of the cytokines IFN-gamma, IL-4 and IL-17 in the cocultured supernatants of 16HBE14o- cells and T cells were determined by ELISA according to the manufacturer's protocols (Sigma, St. Louis, MO, USA).

**Figure 3:**
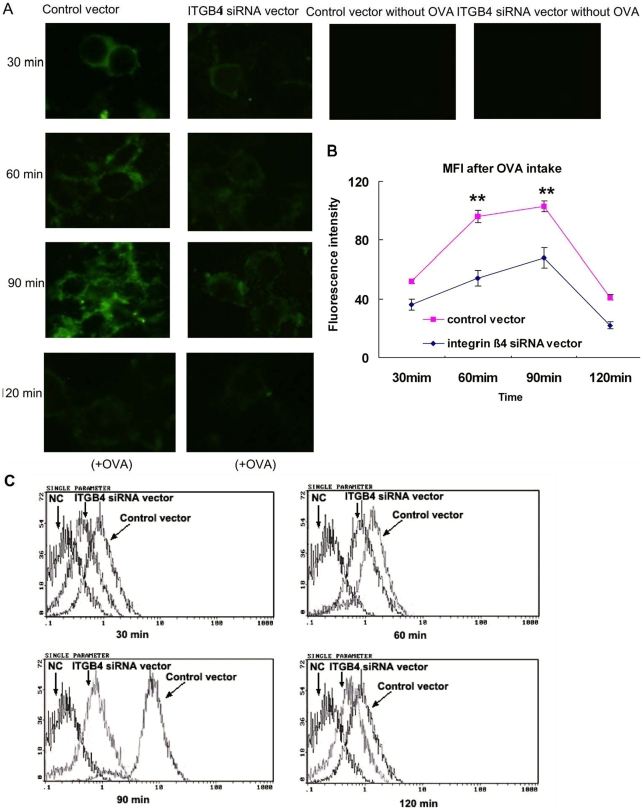
Antigen trafficking was surveyed after ITGB4 was silenced. (A) Control transfected 16HBE14o- cells and ITGB4-silenced cells were grown on coverslips, pulsed with FITC-labeled OVA, washed at different time points, and analyzed through laser confocal microscopy. (B) Quantification of staining intensity in figure 3A. (C) Representative histogranms showing the fluorescence intensity of 16HBE14o- cells from different groups after plused with FITC-labeled OVA. The antigen trafficking ability was determined according to flow cytometry detection at all time points.

### Flow cytometry Assay

To detect the B7 homolog and HLA-DR expression on 16HBE14o- cells after ITGB4 was silenced, the monoclonal antibodies used for flow cytometry were as follows: monoclonal antibody anti-human B7-H1 (Ebioscience, San Diego, CA), B7-H2 (Ebioscience, San Diego, CA), B7-H3 (Ebioscience, San Diego, CA), B7-DC (Ebioscience, San Diego, CA), HLA-DR (Ebioscience, San Diego, CA). Typically, 5–10×10^4^ cells from different groups were incubated in 60 µl of PBS/0.2% BSA/0.1% NaN3 containing saturating concentrations of each monoclonal antibody on ice for 30 min. The cells were then washed and resuspended in the buffer, and immediately analyzed with a FACS Calibur flow cytometer.

**Figure 4:**
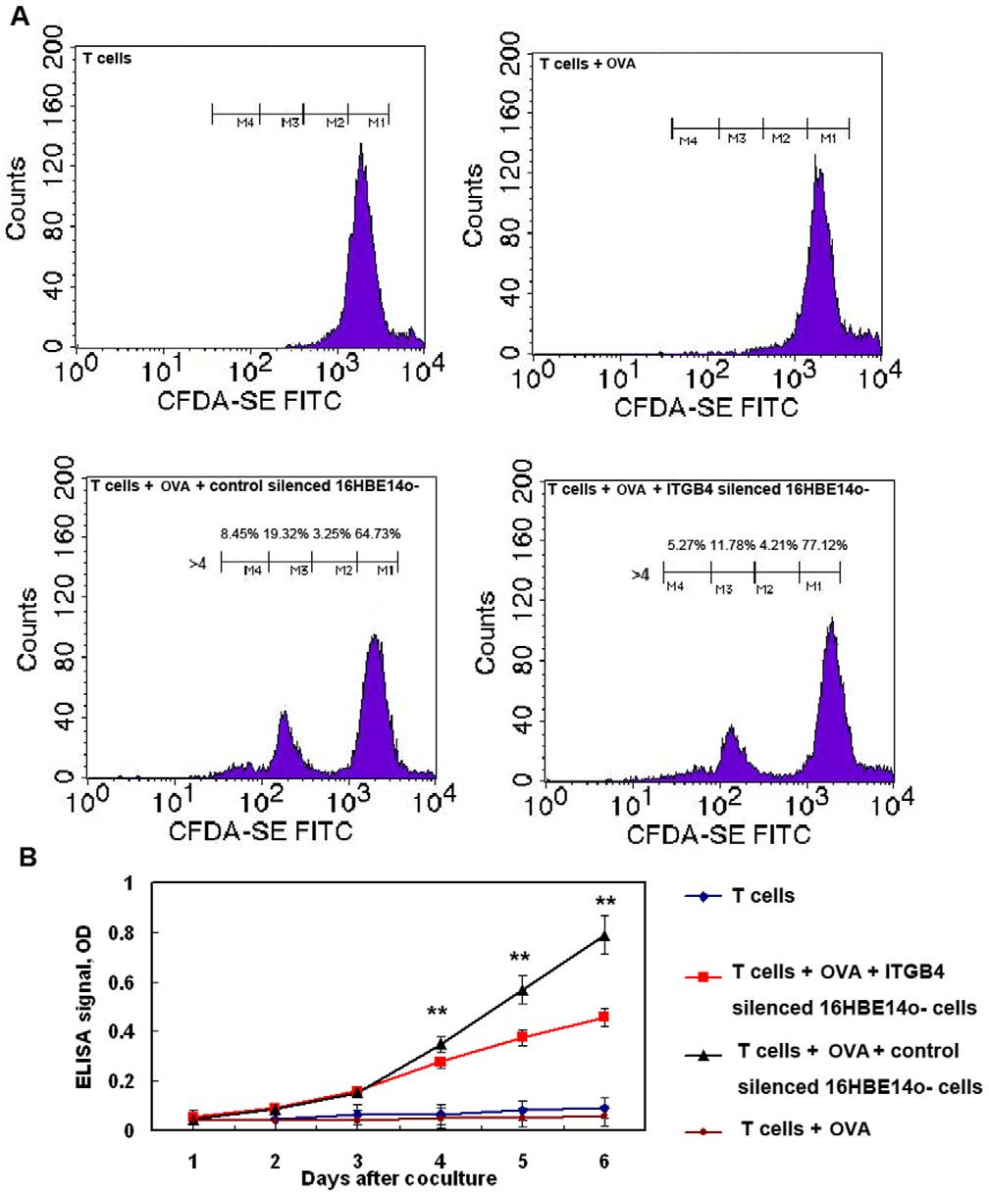
T cell proliferation was observed after coculturing T cells with control transfected 16HBE14o- cells or ITGB4-silenced cells. (A) On the basis of decreased CSFE staining, T cell proliferation are shown as percentage of proliferating cells, and are representative of one experiment repeated six times. Percentage of dividing cells (cells that have undergone 1 division) is shown for control silenced 16HBE14o- cells +T cells and ITGB4 silenced 16HBE14o- cells +T cells cocultures. Proportion of original population induced to divide after stimulation was determined by measuring the number of events in each cell division. (B) Meanwhile, T cell proliferation was assessed by a BrdU incorporation assay after coculture. The data are expressed as the mean (±SEM) values of the specific absorbance from 10 independent experiments.

In addition, the staining of the intracellular cytokines after T cell activation was also measured by flow cytometry. Briefly, T cells previously co-cultured with control vector transfected 16HBE14o- cells or with ITGB4 silenced 16HBE14o- cells were restimulated for 4 h at 37°C in 10%CO_2_ with phorbol ester (50 ng/ml; Sigma), ionomycin (1 mg/ml; Sigma) and GolgiStop (1 ml per 1.5 ml; BD Bioscience).The Cytofix/Cytoperm buffer set (BD) was used for intracellular staining. Cells were fixed and treated with permeabilization buffer according to the manufacturer's instructions and stained with corresponding fluorescence-labeled antibodies (BD). Acquisition was performed with CellQuest software on a FACS Caliber flow cytometry

**Figure 5:**
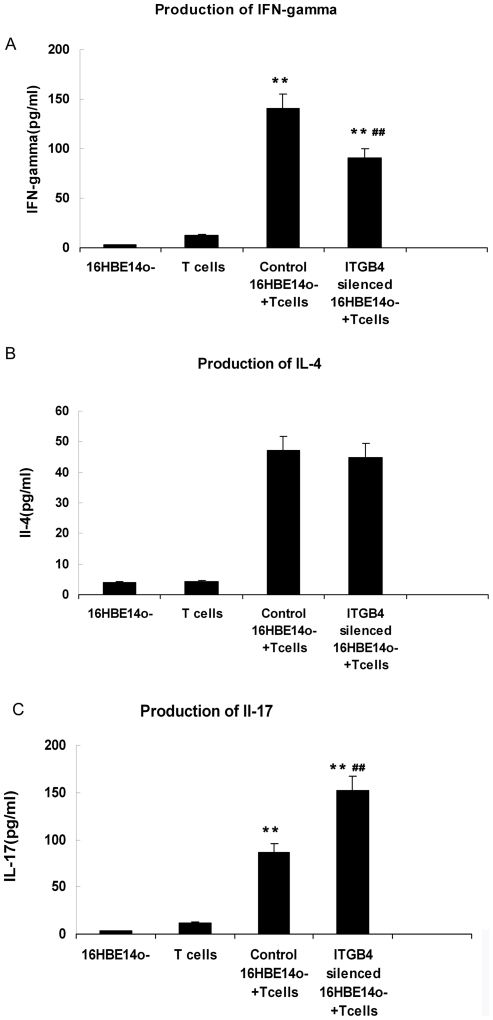
Cytokine concentrations in supernatants of the 16HBE14o- cells and T cells cocultures were quantified by ELISA. The graphs show mean (±SEM) values from a representative experiment of six independent experiments. The content of (A) IFN-gamma, (B) IL-4 and (C) IL-17 was performed respectively.

### Statistical analysis

All experiments were run at least five times. All data are presented as the mean ±standard error of the mean (SEM). For all values, the differences were considered significant when *P<0.05 or **P<0.01. The unpaired t test was used for comparison between two groups. Comparisons of the differences between the ITGB4-silenced group and control groups were performed by means of one-way ANOVA, followed by Dunnett's or LSD's post-hoc test.

**Figure 6:**
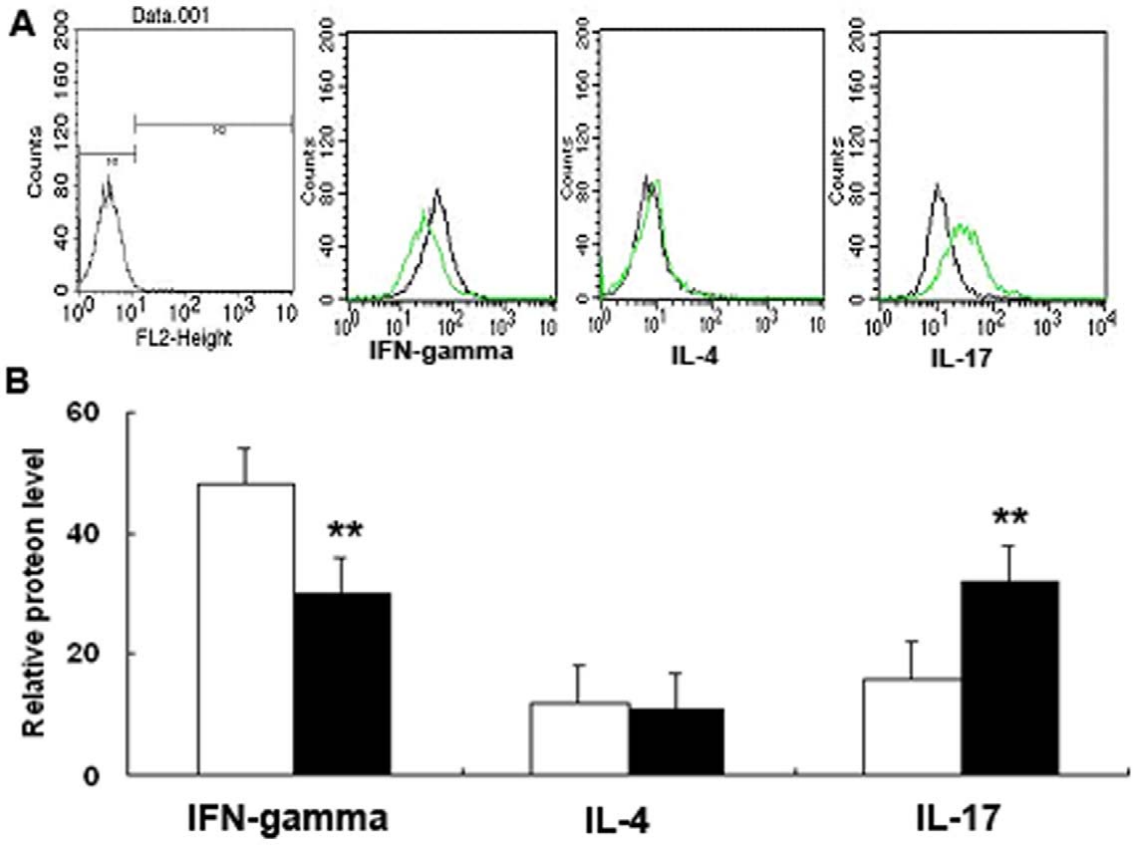
Flow cytometry analysis of the intracellular expression of IFN-gamma, IL-4 and IL-17 at CD4^+^ T cells after coculturing. (A) CD4^+^ T cells from different coculturing groups were stained with specific antibodies to IFN-gamma, IL-4 and IL-17 as described in materials and methods. The histograms shown are from a representative results of six independent experiments are shown. (B) The mean relative fluorescence intensity of intracellular cytokine was quantified after ITGB4 silencing.

## Results

### ITGB4 mRNA and protein expression was effectively silenced by ITGB4 siRNA lentivirus vector

The silencing efficiency on ITGB4 mRNA and protein expression was detected by real time PCR and western blotting. The ITGB4 siRNA lentivirus vector and control lentivirus vector were prepared and transfected into 16HBE14o- cells. Three days after transfection, real time PCR results showed that ITGB4 mRNA was inhibited obviously (**P<0.01, n = 6) (Figure 1.A). Meanwhile, the ITGB4 siRNA virus vector could inhibit ITGB4 protein expression significantly (P = 0.0006, n = 6, ANOVA) (Figure 1.B). More than 75% ITGB4 protein expression was inhibited in the 16HBE14o- cells transfected with ITGB4 siRNA virus vector (Figure 1.C). While compared with the control siRNA vector, there was no significant influence on the ITGB4 protein expression (P>0.5, n = 6, ANOVA) (Figure 1.C). We observed no obvious changes in cell confluence or morphology.

**Figure 7:**
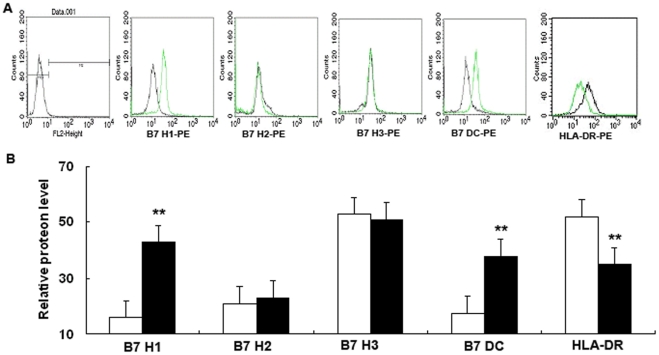
Flow cytometric analysis of cells surface expression of HLA-DR and B7 homologs in 16HBE14o- cells after ITGB4 silencing. (A) Cells from different groups were stained with specific antibodies to indicate HLA-DR and B7 homologs expression as described in materials and methods. The histograms shown are from a representative results of six independent experiments are shown. (B) The mean relative fluorescence intensity of B7 homologs was quantified after ITGB4 silencing.

### Colocalization of Ag with HLA–DR

As respiratory epithelial cells express MHC class II antigens, and in our study induced OVA-specific T-cell proliferation in accord with previously reported data, we wanted to determine whether the endocytosed OVA followed a class II trafficking pattern. We colocalized Dylight 549-labeled anti-HLA–DR antibody with FITC-labeled OVA (red+green = yellow) in 16HBE14o- cells from different groups, using laser confocal microscopy (Figure 2). Colocalization occurred optimally 90 min after OVA pulsing which was diminished 120 min later. These results demonstrate that OVA uptake by 16HBE14o- cells was an active process and was not due to passive diffusion. In addition, FITC labeled OVA, is endocytosed by 16HBE14o- and follows a class II processing pathway.

### FITC-OVA trafficking ability was downregulated in ITGB4-silenced 16HBE14o- cells

By dynamic recording of the OVA fluorescence intensity at different time points, we observed the antigen presentation ability of 16HBE14o- cells. After FITC-labeled OVA was plused with the 16HBE14o- cells, speckled green fluorescence signals could be observed from 30 min. Then, the fluorescence signals increased at 60 min and maximally expressed at 90 min which persisted for up to 120 min. For the ITGB4 silenced group, the uptake of FITC-labeled OVA was decreased obviously (Figure 3.A). In contrast to the conspicuous punctuate staining observed after pulsing with FITC-labeled OVA in control transfected cells, there is a noticeable decrease of fluorescence staining intensity at the same time points in the ITGB4-silenced cells by laser confocal microscopy detection(**P<0.01, n = 6) (Figure 3.B).

To better quantify the differences in OVA uptake after ITGB4 silencing, the mean fluorescence intensity within bronchial epithelial cells at all time points was instantaneously assessed by flow cytometry (Figure 3.C). The 16HBE14o- cells were pulsed with FITC-labeled OVA and collected at all tested time points to detect by flow cytometry. The flow cytometry results demonstrated that the mean fluorescence intensity of 16HBE14o- cells was degraded significantly when ITGB4 was silenced. Therefore, the above results indicated that the antigen uptake ability in 16HBE14o- cells was downregulated when ITGB4 was silenced.

### Comparison of T cell proliferation induced by control 16HBE14o- cells and ITGB4-silenced 16HBE14o- cells

To further investigate the interaction between airway epithelial cells and T cells and to better define the role of 16HBE14o- cells in antigen presentation, we used CFSE staining to assess proliferation of T cells after 16HBE14o- cells and T cells coculture. Experiments were performed using seven different specimens, and the results were examined to ensure that patient variability did not affect the results. After 3 days of coculture, proliferating T cells represented 10–30% of all T cells. In contrast, nonactivated T cells (T cells cultured alone or T cells cultured with OVA) did not divide. Analysis of the CFSE staining showed that the majority of these proliferating cells undergone about three divisions. Percentage of dividing cells is shown for different coculture groups. Our results showed that the ratio of proliferated T cell is obviously decreased in ITGB4 knockdown group compared with control group (20.86%±2.51% vs.32.22%±3.12%,**P<0.01, n = 6) (Figure 4.A). By recording the percentage of dividing cells, we also found that there is a distinguished difference for the ratio in two division cell group (19.57%±2.72% vs. 11.71%±3.45%, **P<0.01, n = 6) and three division cell group (8.45%±3.21% vs 5.69%±2.81%, **P<0.01, n = 6) between the control coculture group and the ITGB4 knockdown coculture group. At the same time, the proliferation of T cells was detected by BrdU-incorporation ELISA. Compared with control group, the number of T cells decreased significantly from the first day to the fifth day after coculture in the ITGB4 silenced group (**P<0.01, n = 10) (Figure 4.B). In addition, the suppression effect became more and more obvious along with the days of coclture.

### Cytokines produced by T cells after stimulation with ITGB4-silenced 16HBE14o- cells

We also determined cytokine (IFN-gamma, IL-4 and IL-17) production as measured by ELISA in supernatant from 16HBE14o- cells and T cells coculture for 3 days. IFN-gamma, IL-4 and IL-17 are typical T cells cytokine that can be used to represent different T cell derivation subpopulation after T cells proliferation. Our results showed that T cells cocultured with 16HBE14o- cells produced IFN-gamma and IL-17, but little IL-4. After ITGB4 silencing, the production of IFN-gamma was decreased (**P<0.01, n = 10, Figure 5.A), IL-17 was increased (**P<0.01, n = 10, Figure 5.C). But there was no obvious difference in IL-4 production (P>0.5, Figure 5.B).

To characterize the source of cytokine production at the cellular level, we simultaneously detect intracellular cytokine staining after couture (Figure 6.A). As expected, in the ITGB4 silencing coculture group, IFN-gamma was decreased (48.6±2.9 vs. 29.2±4.3, P<0.01, n = 6), IL-17 was increased (16.3±2.1 vs. 36.8±3.1, **P<0.01, n = 6). Also, we found little production of IL-4 by all coculturing groups 11.3±0.9 vs 12.7±1.2 P>0.5, n = 6) (Figure 6.B).

### Surface protein expression of B7 homolog and HLA-DR proteins on 16HBE14o- cells after ITGB4 silencing

We set out to extend previous studies to determine whether the expression of B7 homologs and HLA-DR on 16HBE14o- cells was altered after ITGB4 silencing (Figure 7.A). Our results showed that 16HBE14o- cells displayed spontaneously surface expression of B7H1, B7H2, B7H3 and B7DC. And the relative order of B7 homolog family surface expression: B7-H3>B7-H1 » B7-DC ∼ B7-H2. After ITGB4 was silenced, the expression of B7H1 (21.6±2.4 vs 53.7%±2.9, **P<0.01, n = 6) and B7DC (17.5±1.5 vs 33.7±2.2, **P<0.01, n = 6) both increased obviously. But, ITGB4 silencing has no effect on the expression of B7H2 (19±1.5 vs 17.9±1.2, P>0.1, n = 6) and B7H3 (74±2.8 vs 71±3.2, P>0.1, n = 6). As for HLA-DR, our results showed that the expression of HLA-DR was decreased after ITGB4 silencing (55.3±1.7 vs 35.9±3.4, **P<0.01, n = 6) (Figure 7.B).

## Discussion

Allergy immune disorder diseases like asthma are characterized by Th2-mediated inflammation and structural disruption of airway epithelia cells. In these diseases, an increase in Th2 cell infiltration and Th2 cytokine expression is induced after allergy exposure [1]. However, the intrinsic molecular mechanism underlying this Th2 inflammation bias is not fully understood. Previous studies demonstrate that airway epithelial cells engage in the initiation and regulation of inflammation and immune responses through production of cytokines and chemokines and others [18], [19]. Recent studies further showed that bronchial epithelial cells are capable of antigen presentation and thus can regulate the local airway immune cells and inflammation reactions [8], [9]. Especially, T cells can be effectively activated by airway epithelial cells through antigen presentation after allergen exposure on the airway.

The airway epithelial barrier is often disrupted in asthma patients, with evidence for shedding of airway epithelial cells and disparity expression of genes. Our previous study demonstrated that ITGB4 is downregulated in asthma airway epithelial cells, which may result in decreased wound repair and anti-oxidation ability [11], [12]. In this current study, our results demonstrated for the first time that downregulation of ITGB4 blocked the process of antigen trafficking which indicated that the ITGB4-silenced cells are damaged cells and cannot maintain the normal function of monitoring outer allergens and present the foreign antigen to local T cells. As effective APCs, the recognition and uptake of outside allergens is the first step for the local immune homeostasis [20]. Furthermore, the uptake and presentation of antigen by APCs has a close relationship to the activation and differentiation of local T cells [1], [21]. Therefore, we examined the influence of ITGB4 silencing on local airway T cells inflammation. By CFSE staining and BrdU-incorporation assay, we found that the proliferation of T cells was inhibited after coculturing with ITGB4-silenced 16HBE14o- cells. At the same time, T cell-derived IFN-gamma was reduced; IL-17 was increased by ELISA detection and intercellular cytokine staining. It is well known that Th2 cells orchestrate allergy-induced asthmatic inflammatory responses, and the main immune abnormalities are caused by Th2 cytokines, such as IL-4 and IL-5, which induce eosinophil infiltration and airway hyperresponsiveness [4], [7]. On the contrary, IFN-gamma, a Th1 cytokine, reduces airway inflammation and airway hyperesponsiveness in asthma models and clinical traits [22], [23]. Besides, Th17 cells are a recently described effector CD4^+^ T cell subset characterized by the production of IL-17, which have been implicated in the pathogenesis of several autoimmune diseases [24]. IL-17 promotes neutrophil production and chemotaxis via multiple factors which might play a prominent role in the pathogenesis of asthma [25], [26]. Increasing amounts of papers suggested that airway local immune inflammation is a consequence of the actions of many cells and signaling events [4], [21], [27]. Antigen presentation by airway epithelial cells is an effective way to regulate T cell differentiation. The regulation of antigen presentation after ITGB4 silencing would have a close relationship to the asthma airway inflammation. Thus, our results verified the influence of Th2 inflammation induction by ITGB4 low expression on asthma airway epithelial cells. It provided one possible pathway which is correlated to T cell activation and Th2 inflammation bias in the asthma airway mucosa.

Antigen presenting cells are charactered in part by the presence of specific cell surface immune molecules that allow interaction with the T-cell receptor complex. The most well known cell surface protein is the MHC II molecules. We evaluate HLA-DR expression after ITGB4 silencing and find that HLA-DR expression was depressed after ITGB4 was silenced. HLA-DR has attracted considerable interest due to its role in antigen presentation [28]. More importantly, maturation of dendritic cells also results in an increase in HLA-DR expression and the expression of HLA-DR is tightly regulated by inflammatory cytokines[29]. As HLA-DR is a key protein for antigen presentation processes, our results indicated that the decreased antigen presentation ability after ITGB4 silencing partly result from the depressed expression of HLA-DR.

To further explore the possible mechanism for the altered T cells derivation after ITGB4 silence. We investigated the B7 homologs expression after ITGB4 silencing. The flow cytometry results showed that both B7H1 and B7DC was induced after ITGB4 silencing, but no obvious difference was detected for B7H2 and B7H3. It is well known that the optimal activation of T cells requires both costimulation [30] and TCR (T cell receptor) engagement [8], [9]. Antigen presentation in the absence of costimulation may lead to T cell anergy. When the antigen-specific T cells recognize the anigen peptide complex, the following T cell responses are critically affected by the simultaneous signaling operated through B7 costimulatory molecules. Both B7-H2 and B7-H3 are thought to be positive costimulatory ligands in that engagement results in activation of T cell function. Engagement of B7-H2 results in activation of T-helper memory cells with a bias toward Th2 cytokine production, such as IL-4 and IL-13 [31]. Engagement B7-H3 ligands results in proliferation T cells, a bias toward Th1 cytokine production, and primary cytotoxic T cell activation[32]. B7H1 and B7DC are ligands for programmed death-1 (PD-1), which is expressed on activated T cells. Colligation of PD-1 and the T cell receptor leads to the rapid phosphorylation of SHP-2, a phosphatase that inhibited the T cell activation [33], [34]. Given that the PD-1 ligation delivered an inhibitory signal to T cell activity, the upregulation of B7H1 and B7DC after ITGB4 silencing may be associated with T cells proliferation and derivation after allergy exposure. We also blocked the B7H1 and B7DC action with neutralizing antibodies to evaluate the impact on T cell proliferation. The results show that the T cell proliferation was promoted, which are consistent with the previous studies [33], [34]. However, the internal regulatory mechanisms between the T cells inflammation derivation and the B7 family expression are still unclear and more work is needed to explain them.

In light of the data in this work, we find that airway epithelial cells, which constitutively express B7 costimulatory molecules, can regulate the proliferation and differentiation of T cells in the airways by antigen presentation processes. In addition, silencing of ITGB4 in asthma airway epithelial cells led to impaired antigen presentation. In turn, the proliferation of T cells was inhibited, and production of IFN-gamma was decreased, IL-17 was increased, which may relate to Th2 inflammation bias and neutrophil production on asthma airway. These data partly elucidate the role that epithelial cells play in the inflammation phenomenon of asthma and bring some new useful clues to our understanding of the pathogenesis of immune surveillance and inflammation responses in asthma.
